# The real threat of swimmers’ itch in anthropogenic recreational water body of the Polish Lowland

**DOI:** 10.1007/s00436-016-5060-z

**Published:** 2016-04-15

**Authors:** Anna Marszewska, Anna Cichy, Tomasz Heese, Elżbieta Żbikowska

**Affiliations:** Department of Invertebrate Zoology, Faculty of Biology and Environment Protection, Nicolas Copernicus University in Toruń, Toruń, Poland; Department of Environmental Biology, Faculty of Civil Engineering, Environmental and Geodetic Sciences, Koszalin University of Technology, Koszalin, Poland

**Keywords:** Anthropogenic reservoir, Swimmers’ itch, Bird schistosome, Cercariae, *Planorbarius corneus*, *Radix* spp.

## Abstract

After numerous reports the local press about the “stinging water” in created on the Dzierżęcinka River—Water Valley reservoir and recognizing in bathers the symptoms of swimmers’ itch, environmental study on the presence of bird schistosome larvae in snail hosts was conducted. Snails belonging to Lymnaeidae and Planorbidae were collected at two sites: (i) part of anthropogenic reservoir (192 individuals) and (ii) the river part (37 individuals). Higher prevalence of Digenea was observed in snail populations living in Water Valley (29.8 %) compared to Dzierżęcinka River (21.3 %). The larvae of bird schistosomes were recorded in both localities in 1.8 % of collected snails. The prevalence of bird schistosomes reached 2.9 % in *Planorbarius corneus*, 2.8 % in *Radix auricularia*, and 5.9 % in *Radix balthica/labiata*. Laboratory tests have shown that at 19 °C the number of bird schistosome cercariae released from snail hosts significantly exceeded the number of cercariae of other identified Digenea species. It is worth underlining that despite the low prevalence of bird schistosomes, the high number of released cercariae was sufficient to create a real threat of swimmers’ itch in bathers. As indicated by the example presented, anthropogenic reservoirs create excellent conditions for Digenea species including bird schistosomes. In view of the real risk of people using the waters, tests on presence of the parasites in snail hosts should be included to the standard procedure of security control in bathing places.

## Introduction

Cercarial dermatitis called swimmers’ itch has been listed for several years in human populations around the world (Cort 1936; Hunter et al. 1949; Jarcho and van Burkalow 1952; Macy 1952; Hoeffler 1974; Leedom and Short 1981; Eklu–Natey et al. 1985; Blankespoor and Reimink 1988; Loken et al. 1995; Pilz et al. 1995; Lindblade 1998; Kolářová et al. 1999). It reveals an allergic skin reaction (Kolářová et al. 2010) in the form of painful, then itchy lumpy rash (Żbikowska et al. 2002). It occurs in people bathing or wading in ponds populated by snails infected with bird schistosome larvae. Lumpy skin lesions resembling early stage of chickenpox, is a result of penetration by cercariae of bird schistosomes, and the number and size of bubbles depend on the number of penetrating larvae (Żbikowska 2003).

Snails play a key role as first intermediate hosts in the life cycle of bird schistosomes. Miracydium enters the mollusk, then in hepatopancreas transforms into sporocyst, inside which after 6–7 weeks numerous furcocercariae invasive for vertebrates emmerge (Amen and Meuleman 1992). Cercariae abandon host snail and penetrate the webbed feet of waterfowl, transforming into schistosomulae. In ventral organs of bird host, parasites mature and reproduce sexually (Soldánová et al. 2013). However, bird schistosomes are not capable of sexual maturity in man, some authors suspect the possibility of schistosomulae occurrence inside human organs (Horák and Kolářová 2001; Olivier 1953). This point of view is based on facts that appearing dermatitis in a person infected can be accompanied by further symptoms such as nausea, diarrhea, swollen glands, insomnia, fever (Horák et al. 2002; Żbikowska et al. 2002), or even anaphylactic shock and disorders of the respiratory system (Bayssade–Dufour et al. 2001). Additionally, the results of studies conducted on experimentally invaded mammals showed presence of schistosomulae in their lungs (Horák and Kolářová 2001; Haas and Pietsch 1991; Appleton and Brock 1986), liver, kidney, heart or intestine (Haas and Pietsch 1991; Horák and Kolářová 2000). A particularly interesting finding of bird schistosome larvae in the lungs of rhesus monkeys (Olivier 1953)—most similar research model for *Homo sapiens*—should be emphasized.

The potential risk of human invasion is increased by the fact that bird schistosomes quoted around the world including several taxa of hosts (Liu 2012). Some species belonging to the so-called “nasal schistosomes” are considered to be particularly dangerous because they have a high affinity for the central nervous system (brain and spinal cord) (Horák et al. 1999; Kolářová et al. 2001; Hrádková and Horák 2002). Kouřilová et al. (2004) and Lichtenbergová et al. (2011) observed a locomotion disorder in mice experimentally invaded by nasal bird schistosome *Trichobilharzia regenti*, very similar to those in typical bird host. No conclusive data on the fate of penetrating human skin cercariae, along with the growing number of documented invasion by bird schistosomes in people around the world (Marie et al. 2015; Gohardehi et al. 2013; Valdovinos and Balboa 2008; Rao et al. 2007), may negatively affect the local tourism economy (Horák et al. 2015), and although for that reason, the problem of these parasites invasion needs further study.

Swimmers’ itch in Europe is mostly recorded in anthropogenic basins and eutrophic lakes (Soldánová et al. 2013). Risk assessment and preventive measures are usually introduced where the problem swimmers’ itch is the fact (Jouet et al. 2008; Verbrugge et al. 2004; Caumes et al. 2003; Lévesque et al. 2002; Chamot et al. 1998). One of the examples—French lake Annecy—indicates the need for the earlier prevention preceding the planned changes in environment. This attractively landscaped downtown area has become the place of numerous cases of dermatitis caused by bird schistosomes (Caumes et al. 2003). The high number of reported cases in humans was associated with the increase in population of *Radix* sp. and the creation of an artificial bird-island in the lake (Jouet et al. 2008). Human intervention in the ecosystem of the Lake Annecy caused unforeseen consequences, the manifestation of which was increasing cases of swimmers’ itch.

In Poland, in response to the needs of local communities, authorities started to create inner-city baths, being an interesting alternative to indoor swimming pools and water parks in the summer. Expanding river and streams beds and damming in the areas without earlier bathing places, creates good conditions for penetration both—the elements of wild ecosystem and human habitat. One of the adverse effects of changes in the environment may be the emergence of swimmers’ itch in people using new recreational waters. In this paper, we present an analysis of coincidence between water-derivate dermatitis and etiological factors of swimmers’ itch in the area of anthropogenic reservoir in the Polish Lowland. Our thesis concerns potential health risks accompanied by water ecosystem transformation and addresses the need to take those risks into account in future hydro plans.

## Material and methods

### Description of the area

Multifunctional reservoir on the Dzierżęcinka River was opened on October 14th, 2013, in the place of the retention basin functioning before World War II, much smaller than the current one. Covering the area of 6.05–7.08 ha, the reservoir is 800-m long and reaches 150 m in its widest point. It is 2 to 2.5-m deep and may take from 118 000 to 152 000 m^3^ of water. In addition to the role of water storage and fire protection, it is a recreational attraction for locals and tourists, called the Water Valley. The hydro project is one of five steps implemented within the framework: “Protection of the Jamno Lake flood basin and revitalization of the Dzierżęcinka River – protection areas of Koszalin.”

The river with a length of 29.3 km has its source in the west of the village Kliszno. In the initial run, below the village Manowo, it flows in artificially created riverbed, connecting with the Lubiatowskie Lake. From the outflow of the lake the river flows in the XIII century canal, then continues through the city of Koszalin in a deep glacial valley, supplying the Pond Castle (1.5 ha) in the Park of the Pomeranian Dukes. After leaving the northern part of the city the river flows into the coastal lake Jamno.

Six years ago, before the launch of a revitalization plan, the Dzierżęcinka River carried waters of very low quality. The main reason was illegal sewage pollution of the river. The actions taken by the local authorities have contributed not only to the improvement of esthetic qualities of the river but also to some positive changes in the development of biocenosis. Nowadays, fishing sources report the increase in biological diversity of fish populations—occurrence of tench, crucian carp, roach, ide, and also pike and eels. Additionally, damming and the creation of the reservoir have caused some waterfowl of the Park of Pomeranian Dukes move into the area of the Water Valley. The current swimming pool is a picturesque place where the processes within the biocenosis intersect with the needs of active rest of people. A safe area has been separated for children and young people to play in, comprising a small canoe dock, a beach area, and a walking zone along the banks of the reservoir.

### Medical case description

**Fig. 1:**
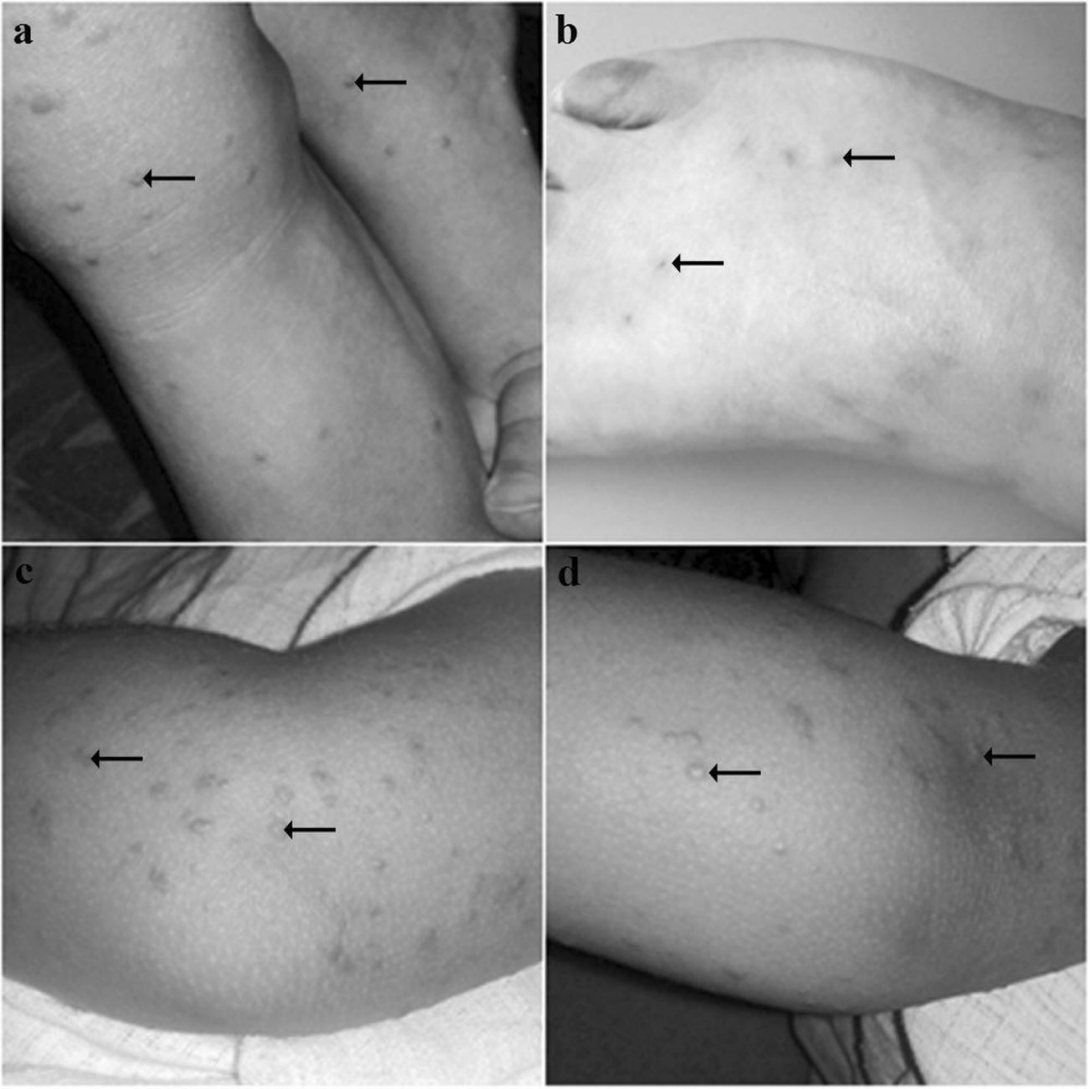
Swimmers’ itch on: **a** The legs of 3-year girl 2 weeks after infection, **b** The leg of adult man 2 weeks after infection, **c** The forearms of 3-year girl 2 weeks after infection, **d** The forearms of 3-year girl 4 weeks after infection

In the summer 2015, on June 27th a 3-year girl went to the doctor’s surgery because of a sore throat, with body temperature elevated up to 38 °C and the symptoms of swimmers’ itch on the skin of both legs (Fig. 1a) and forearms (Fig. 1c) continuing for 2 weeks. The dermatitis consisted of minor lumps, resembling the ones in chickenpox. In the treatment, fenistil and pimafucort cream to the local lubrication was applied for weeks—the child went to the doctor again, because the itching intensified, and the changes were not reversible; the bumps on the forearms were still noticed (Fig. 1d). In addition, the child developed catarrh and catarrhal changes of the nasal mucosa. After dermatological consulting, the treatment was maintained for the next weeks and then the lesions gradually disappeared.

Medical interview shows that skin symptoms in a child appeared a few hours after bathing in the Water Valley reservoir. At the same time, a similar rash appeared in an adult caregiver, bathing with the child in the basin described above. In the case of the adult, the same treatment was used, the effectiveness of which was already observed after 2 weeks of application of medication. The changes in the skin gradually underwent exfoliation (Fig. 1b).

During the summer season of 2015, more than ten cases of similar rashes caused by the “stinging water” in the Water Valley was reported (personal information). Local authorities and sanitary service decided to examine water samples for the presence of toxic bacteria and blue-green algae. The tests carried out by the sanitary station and the Inspectorate of Environmental Protection did not reveal the presence of the toxic microorganisms in water (Rejestr Stacji Sanitarno–Epidemiologicznej w Koszalinie 2015).

### Parasitological diagnosis of potential intermediate hosts of bird schistosomes

Material for parasitological study were pulmonate snails: *Lymnaea stagnalis*, *Radix auricularia*, *Radix balthica/labiata*, and *Planorbarius corneus*, which can play a role of intermediate hosts of bird schistosomes. Snails were collected in August 2015, in two localities—(i) the Water Valley (54°10′45″N, 16°12′31″E), and for comparison in (ii) parts of the Dzierżęcinka River running through the Park of Pomeranian Dukes (54°11′13″N, 16°11′15″E). Snails were collected from plants and the bottom of reservoir and the river by hand or by using metal sieves.

Taxonomic affiliation of collected snails was verified on the basis of morphological data (Piechocki 1979), and anatomical features of the reproductive system—in the case of the genus Radix (Jackiewicz 2000). All collected snails were individually kept in culture beakers with conditioned tap water in the incubator (SANYO) at 19 °C and natural photoperiod. The culture temperature was determined based on the results of previous studies on thermal preferences of snails (Żbikowska 2005). Diagnostics of parasite presence was carried out by a non-invasive method, and Digenea species were recognized on cercariae morphology according to Faltýnková et al. (2007, 2008). Every day, each snail was placed for 1 h in a beaker with a small amount of water under a light source. Under these conditions, snails with patent invasion released cercariae. Snails were replaced into culture beakers filled with fresh water, and cercariae in small beakers were fixed with 75 % ethanol then counted by quantitative method in a Sedgwick-Rafter chamber. The survey was repeated until the death of all snails. Dead animals were necropsied for checking their invasion status. At the end of the experiment, the obtained data were divided into the groups on the basis of invasion status of snails—non-infected and infected with different parasite species. Snails’ lifespan duration and number of cercariae released from naturally invaded hosts were analyzed.

### Statistical analysis

A Chi-square test of contingency table was used to compare the number of infected and non-infected snails collected in both sampled localities. For further analysis, concerning lifespan of snails and the number of released cercariae only individuals of the same snail genus were used—naturally invaded with bird schistosomes, hosts of other Strigeida species and non-infected snails. Only the groups that reached at least three specimens were compared.

The average number of Strigeida species cercariae (furcocercariae per snail host) was calculated, and the results were analyzed by one-way ANOVA (factor: parasite species), followed by post-hoc Tukey test. The same type of analysis was used to compare an average lifespan duration of the studied snail groups.

The term prevalence (%) was used for the description of one snail species invaded by bird schistosomes, whereas the term infection (%)—as a proportion of hosts infected by specific parasite species in relation to all invaded snails.

## Results

**Fig. 2:**
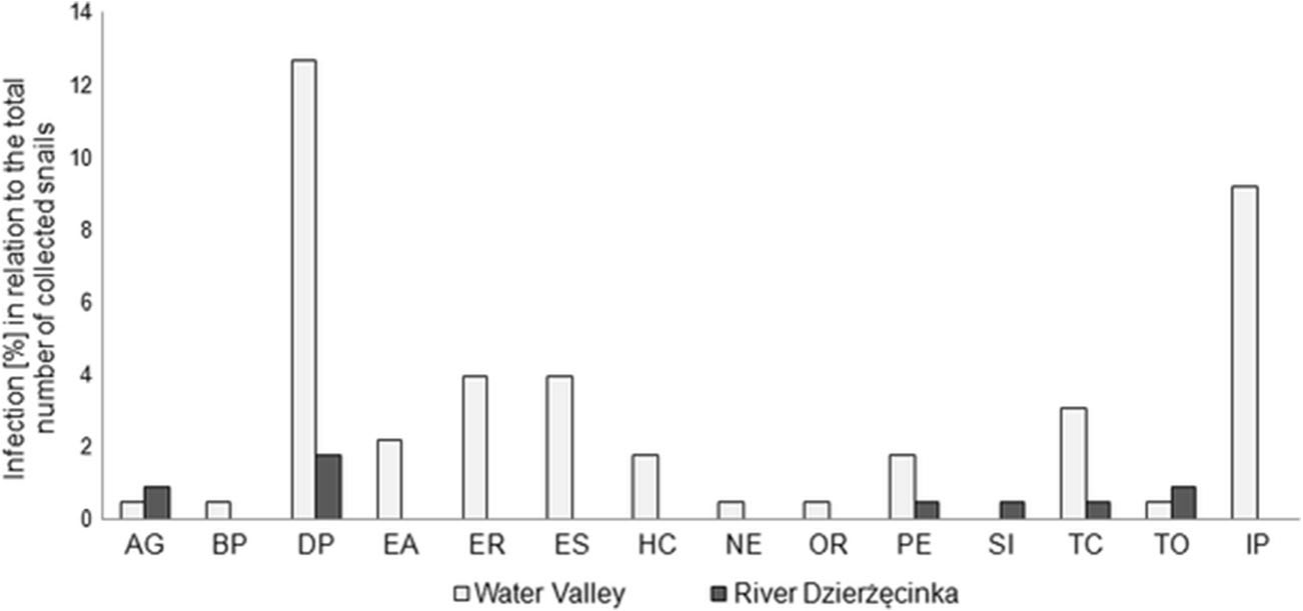
Infection of snails by Digenea larvae in collected samples. Parasites—Srigeida: *AG Apatemon gracilis*, *BP Bilharziella polonica*, *DP Diplostomum pseudospathaceum*, *SI Sanguinicola inermis*, *TC Tylodelphys clavata*, *TO Trichobilharzia ocellata*; Echinostomida: *EA Echinoparyphium aconiatum*, *ER Echinostoma revolutum*, *ES Echinostoma spiniferum*, *HC Hypoderaeum conoideum*, *NE Notocotylus ephemera*; Plagiorchiida: *OR Opisthoglyphe ranae*, *PE Plagiorchis elegans*; undiagnosed pre-patent invasion—PI

In total, 229 snails were sampled: 106 individuals of *Lymnaea stagnalis*, 17 *Radix balthica/labiata*, 71 *Radix auricularia*, and 35 *Planorbarius corneus*. In the Park area of the Dzierżęcinka River only 37, while in the Water Valley the remaining 192 individuals were collected. On both studied localities, the same snail species were found. In nearly half of all the collected animals, 94 revealed the presence of Digenea larvae. Among the snails collected in the Water Valley, 29.8 % were naturally invaded by parasites and in the samples from park part of the Dierżęcinka River only 21.3 %. The difference was statistically significant (*χ*2 = 100.25, df = 1, *P* < 0.001). Also the number of diagnosed parasite species found in the snails sampled in the Water Valley was higher. Among the total 14 identified Digenea species, 12 were noted in the Water Valley and only 6 in park locality. Throughout the parasites developing inside the studied snails dominated fluke species producing furcocercariae (Fig. 2).

Larvae of bird schistosomes were recorded in both studied localities (Fig. 2). The snails naturally infected with bird schistosomes accounted for 1.8 % of all collected specimens. In the Water Valley bird schistosome larvae were detected in *P. corneus* (*Bilharziella polonica*) and in *R. auricularia* (*Trichobilharzia* sp.) while in park locality, only *Trichobilharzia* sp. was found but inside two lymnaeids snail species—*R. auricularia* and *R. balthica/labiata*. The prevalence of bird schistosomes in snails’ populations was low and amounted to 2.9 % (in *P. corneus*), 2.8 % (in *R. auricularia*) and 5.9 % (in *R. balthica/labiata*).

There was a statistically significant difference in the lifespan between four groups of *Radix* sp. individuals kept at controlled temperature conditions 19 °C (one-way ANOVA F_3,8_ = 33.17, *p* < 0.001) (Table 1). Those snails at experimental conditions released different, parasite species dependent number of cercariae (one-way ANOVA F_2,6_ = 62.12, *p* < 0.001). Post-hoc tests indicated that amount three furcocercous Digenea species invading *Radix* sp. the most productive were bird schistosomes from complex species *T. ocellata* (Table 1).

**Table 1:** Snail survival and cercariae production in experimental condition: constant temperature and natural photoperiod

*Radix* sp.	Temperature (°C)	Survival avg. (days) (± SE)	Number of emerged cercariae/snail (± SE)
Non–infected	19	23 (±1)	–
With *T. ocellata*	19	12 (±1)*	1657 (±54)a
With *D. pseudospathaceum*	19	11 (±1)*	990 (±110)b
With *T. clavata*	19	8 (±1)*	503 (±35)c

Different letters indicate statistically significant difference between compared values (a/b, *p* < 0.01, b/c, *p* < 0.01, a/c, *p* < 0.001)

*Statistically different from non-infected ones (Tukey test *p* < 0.001)

## Discussion

The lowering of groundwater level and the need for rational management of water resources are the reasons for using the artificial reservoirs in summer season for recreational or educational purpose. The latter function may take an unexpected course of “education on the living organism,” as evidenced by the above case. The occurrence of swimmers’ itch in the child and adult bathers in the artificial reservoir is the first well-documented case in this region, although another one associated with anthropogenic reservoir used for recreation in the world (Chamot et al. 1998; Lindblade 1998; Lévesque et al. 2002; Caumes et al. 2003; Soleng and Mehl 2011).

A medical case presented in this paper fully corresponds to the characteristics of swimmers’ itch (Żbikowska et al. 2002). The location of dermatitis—on the parts of the body that come into contact with the water of basin, the rash, resembling the initial stage of chickenpox, and the time elapsed since the contact with the lake water to the appearance of first symptoms (Liu 2012) indicate the related cercariae etiology of illness. This point of view is additionally underlined by negative results of bacterial and blue-algae tests made in Sanitary Station (Rejestr Stacji Sanitarno–Epidemiologicznej w Koszalinie 2015).

It is particularly noteworthy that most severe symptoms of disease were observed in the child compared with the adult, which is consistent with the reports of other authors who indicated even bronchial reactions in the course of the attack of bird schistosomes in children (Bayssade–Dufour et al. 2001). Moreover, the differences in the course of the reported cases, confirm the individual susceptible of host (depending on the activity of the immune system) (Kolářová et al. 2013).

As follows from the case, a rash in the child and the adult appeared a few hours after the bath in anthropogenic basin. Creating such reservoirs, according to some authors, has a dramatic impact on the environment (Morley 2007). Researchers agree that not all the consequences of the changes can be predicted and therefore they postulate the need for constant monitoring of the environment (Morley 2007; Żbikowski et al. 2007). Artificial reservoirs built on the rivers require special attention. They create new conditions for biota, causing rapid qualitative and quantitative changes in the environment (Poznańska et al. 2009; Żbikowski et al. 2007). Such reservoirs are characterized by increased surface compared to the previous one, attracting numerous species of waterfowl (Morley 2007). The created lagoon is characterized by a slower flow of water and a better developed coastline, thus creating more favorable conditions for many invertebrates e.g. molluscs (Poznańska et al. 2009; Żbikowski et al. 2007). These changes concern both—free living organisms and the accompanying parasites (Morley 2007). The effects of such changes unintentionally may turn against human posing a serious threat to health, affecting children in particular.

Such unforeseen result was the appearance of the real problem of swimmers’ itch in the basin described. Statistical analysis of the data shows that both the intermediate Digenea hosts and the parasites have gained better living conditions in parts of the reservoir as compared to the river. Shallow depth of the river flowing in the park, a high water transparency, and mass colonization by feeding waterfowl were the factors effectively limiting the development of molluscs (van Leeuwen et al. 2012). The number of snails collected in the river accounted for only the sixth part of the whole sample. Despite the similar snail host species composition on both studied localities, there were significant differences concerning diversity and prevalence of parasites.

Good living conditions for the snail development in the reservoir, the presence of waterfowl freely moving between both parts, and recreational use of the Water Valley allowed the invasion of bird schistosome cercariae to the people. A similar coincidence effect of these factors was observed in the reservoirs created on the River Ruhr (Germany), where the prevalence of infection *R. auricularia* by *T. franki* for one of the sampling sites was up 27 % (Soldánová et al. 2010).

In the samples, larvae of bird schistosomes were found in three species of snails, which confirms the favorable conditions for the transmission of these parasites in the environment. Most of the infected snails belonged to the family Lymneidae, considered by Horák et al. (2002) as the source of the main etiological factors of swimmers’ itch in Europe—the larvae of the genus Trichobilharzia. Preliminary studies carried out in parts of the park have allowed a note of the presence of cercariae of complex species *T. ocellata* in individuals *R. balthica/labiata* (*=R. ovata/peregra*) (Gloger and Meier–Brook 2003), which is a host for nasal schistosome *T. regenti* (Lichtenbergová and Horák 2012). After skin penetration, nasal schistosomes migrate through the central nervous system of vertebrate host. This migration causes tissue damage, and also locomotion or orientation disorders in bird or mammals hosts (Horák et al. 1999; Kolářová et al. 2001; Kouřilová et al. 2004; Lichtenbergová et al. 2011). The mere suspicion of the presence of nasal schistosomes in the study area indicates the need for monitoring of parasites in the environment, and determination of the taxonomic affiliation, which requires molecular diagnostics.

The real threat of swimmers’ itch in the basin lies in a dissonance with a low prevalence of bird schistosomes in snail host populations, however the recorded values (2.8–5.9 %) did not differ from the results of other authors conducting research in the regions of frequent occurrence of this kind of dermatosis (Skírnisson and Kolárová 2005; Farahnak and Essalat 2003; Lévesque et al. 2002; Picard and Jousson 2001). The real danger of the invasion can be affected by three important factors: (i) high productivity of cercariae inside snail host, (ii) extended life of the infected snails, and (iii) high survival of invasive cercariae in an environment. This hypothesis was positively verified by the result of Soldánová et al. (2016) and also of current observation (Table 1)—the number of produced bird schistosome cercariae by the same snail species was significantly higher in comparison to other Digenea or our previous laboratory studies (Żbikowska 2004a, b, 2005, 2012)—LT_50%_ of the host survival dependent on temperature and was 32 to 79 days, and cercariae of *T. ocellata* under culture conditions alive even more than 30 h.

Temperature has a significant and direct influence on a life cycle of trematodes (Mas–Coma et al. 2009). In physiological limits, temperature increase accelerates the development of parasites inside ectothermic animals (Kendall and McCullough 1951; Lo and Lee 1996; Poulin 2006), but may also cause an accelerated death of hosts (Żbikowska 2005; Lee and Cheng 1971). These observations were also confirmed in our study, it has been demonstrated that naturally infected snails lived significantly shorter than non-infected ones (Table 1). On the other hand, the increase in ambient temperature can facilitate the transmission of free-living stages of the parasites to next hosts (Poulin 2006), but beyond a certain value their invasiveness can dramatically decrease (Morley and Lewis 2013; Morley et al. 2010). Taking into account the complexity of the effect of temperature on the biology and survival of bird schistosomes and their snail hosts, it can be assumed that in the summer, the density of invasive cercariae in the basin of the Water Valley reached a critical value.

## Conclusion

Not clearly explained the ultimate fate of bird schistosome larvae penetrating human skin, additional symptoms like fever, catarrhal changes in the mucosa and bronchial obstruction in people, especially in infected children, and common presence of these parasites in environment are a strong argument for the inclusion of snail testing for the presence of bird schistosome larvae to the standard control procedure of security in bathing places. Interesting postulate in this field has been put forward by the Danish researchers Christiansen et al. (2016), suggesting the use of molecular diagnostics of pre-patent phase of the bird schistosomes development in snails.

Environmental monitoring and molecular diagnostics of bird schistosome larvae in snail host population from bathing places will be conducted in the coming period of active vegetation.
